# The evolutionary trajectories of *P. aeruginosa* in biofilm and planktonic growth modes exposed to ciprofloxacin: beyond selection of antibiotic resistance

**DOI:** 10.1038/s41522-020-00138-8

**Published:** 2020-07-24

**Authors:** Marwa N. Ahmed, Ahmed Abdelsamad, Tina Wassermann, Andreas Porse, Janna Becker, Morten O. A. Sommer, Niels Høiby, Oana Ciofu

**Affiliations:** 1 Costerton Biofilm Center, Department of Immunology and Microbiology, University of Copenhagen, Copenhagen, Denmark; 2 Department of Microbiology, Faculty of Agriculture, Cairo University, Giza, Egypt; 3 Department of Genetics, Faculty of Agriculture, Cairo University, Giza, Egypt; 4 Department of Clinical Microbiology, Rigshospitalet, Copenhagen, Denmark; 5 Novo Nordisk Foundation Center for Sustainability, Technical University of Denmark, Lyngby, Denmark

**Keywords:** Biofilms, Molecular evolution

## Abstract

Ciprofloxacin (CIP) is used to treat *Pseudomonas aeruginosa* biofilm infections. We showed that the pathways of CIP-resistance development during exposure of biofilms and planktonic *P. aeruginosa* populations to subinhibitory levels of CIP depend on the mode of growth. In the present study, we analyzed CIP-resistant isolates obtained from previous evolution experiments, and we report a variety of evolved phenotypic and genotypic changes that occurred in parallel with the evolution of CIP-resistance. Cross-resistance to beta-lactam antibiotics was associated with mutations in genes involved in cell-wall recycling (*ftsZ*, *murG*); and could also be explained by mutations in the TCA cycle (*sdhA*) genes and in genes involved in arginine catabolism. We found that CIP-exposed isolates that lacked mutations in quorum-sensing genes and acquired mutations in type IV pili genes maintained swarming motility and lost twitching motility, respectively. Evolved CIP-resistant isolates showed high fitness cost in planktonic competition experiments, yet persisted in the biofilm under control conditions, compared with ancestor isolates and had an advantage when exposed to CIP. Their persistence in biofilm competition experiments in spite of their fitness cost in planktonic growth could be explained by their prolonged lag-phase. Interestingly, the set of mutated genes that we identified in these in vitro-evolved CIP-resistant colonies, overlap with a large number of patho-adaptive genes previously reported in *P. aeruginosa* isolates from cystic fibrosis (CF) patients. This suggests that the antibiotic stress is contributing to the bacterial evolution in vivo, and that adaptive laboratory evolution can be used to predict the in vivo evolutionary trajectories.

## Introduction

Sublethal concentrations of antibiotics are present in clinical settings at the infection site in certain tissues as a consequence of limited accessibility as well as in the environment. It has been shown that resistant mutants, even with high MICs can be selected at sublethal antibiotic concentrations^1–3^. In addition, antibiotics at sublethal concentrations are responsible for increasing genetic variation^4^ by means of different pathways involving oxidative stress^5^, by inducing error-prone polymerases mediated by the SOS response^6^, by misbalancing nucleotide metabolism or acting directly on DNA^7^. In this respect, quinolones have been known for years to be mutagenic in bacteria^8^. This implies that antibiotic therapy, in some cases, may have the detrimental side-effect of accelerating the adaptation of pathogens as well as of the commensal strains (suggested to be a major reservoir of resistance).

We have previously reported in a planktonic experimental evolution study conducted on more than 900 generations of *Pseudomonas aeruginosa*, that high-level CIP-resistant mutants with cross-resistance to beta-lactams developed quickly in the presence of a subinhibitory concentration of ciprofloxacin^9^, and a phenotypic shift of the *P. aeruginosa* populations evolved under constant exposure to subinhibitory concentrations of CIP^10^. Compared to the controls (CTRL), the populations evolved in the presence of sublethal concentrations of CIP showed decreased protease activity and swimming motility, higher levels of quorum-sensing (QS) signal molecules and upregulation of denitrification genes^10^. These observations suggested that evolution in the presence of sublethal concentrations of CIP has pleiotropic effects on the bacterial phenotypes and this might promote persistence of the resistant bacterial population.

Persistent infections are caused by biofilm-embedded bacteria which are characterized by multifactorial intrinsic tolerance (phenotypic resistance) to antibiotics^11^. However, several recent studies have demonstrated the supplementary role played by mutational resistance in the survival of biofilms to antibiotic treatment^12,13^.

We have recently investigated the evolution of mutational resistance in *P. aeruginosa* biofilms exposed to subinhibitory concentrations of CIP and showed that the biofilm mode of growth promotes development of low-level mutational resistance^14^ and that this process was accelerated during evolution of a *P. aeruginosa* catalase mutant, as a surrogate of the oxidative stress environment present at the site of chronic infections^15^.

**Fig. 1:**
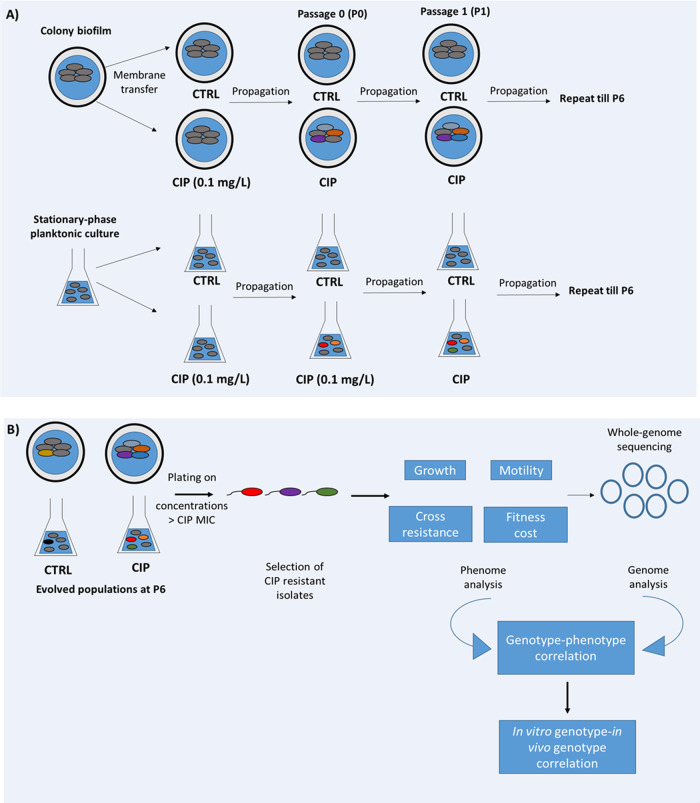
Experimental design of the in vitro *P. aeruginosa* evolution studies and analysis of the evolved isolates. **a** Experimental evolution in colony biofilm and stationary-phase planktonic culture. A colony biofilm was formed on polycarbonate membrane from an overnight culture of *P. aeruginosa* (WT PAO1 or Δ*katA*). In the step called passage 0 (P0), membranes containing 48-h colony biofilms were transferred to fresh LB plates with either CIP or without CIP for 48 h (CTRL). Every 48 h, the colony biofilms were disrupted, and the bacterial populations were used to start new biofilms. The bacterial populations of disrupted CIP biofilms were used to start new colony-biofilms on plates with CIP and the bacterial populations of disrupted CTRL biofilms were used to start new colony-biofilms on LB plates. This was repeated for six passages (P1–P6). **b** Evolved biofilm and planktonic populations (CIP and CTRL) were plated on concentrations higher than the minimum inhibitory concentrations (MIC) of ciprofloxacin. The resistant colonies were collected for phenotypic and genotypic analysis to elucidate the evolutionary trajectories under exposure to subinhibitory concentrations of ciprofloxacin.

The phenotypic shifts that we have previously observed in the experimental evolution of planktonic cultures exposed to sublethal levels of CIP^10^ encouraged us to investigate, the phenotypic and genomic evolution associated with CIP-resistance in biofilms^14,15^. The experimental set-up of the investigation of the phenotypic and genotypic landscape of evolution under exposure to subinhibitory levels of CIP is presented in Fig. 1.

We show here that prolonged exposure of *P. aeruginosa* biofilms to sub-MIC CIP led to mutations which were associated with extensive phenotypic changes. Cross-resistance between CIP and beta-lactam antibiotics such as aztreonam and ceftazidime, differential swarming and twitching motility compared to CTRL, growth curves with a prolonged lag phase, and metabolic shifts toward anaerobic respiration were observed. A fitness cost of CIP resistance in biofilms was observed in the absence of antibiotics. However, non-surprisingly, CIP-resistant colonies had increased fitness when the competition experiments were conducted in the presence of CIP. Overall, our data show that under antibiotic stress, mutational resistance to CIP associates with mutations in several genes that probably play a role in the persistence of biofilms during antibiotic treatment. Interestingly, the set of mutated genes that we identified in these in vitro-evolved CIP-resistant colonies, overlap with a large number of patho-adaptive genes previously reported in *P. aeruginosa* isolates from cystic fibrosis (CF) patients. This is identifying antibiotic stress as an important environmental factor that shapes the bacterial evolution in vivo. It can thus be speculated that this, together with the inflammatory response leads to the so-called chronic phenotype. Chronic phenotype occurs when bacteria become well-adapted to the conditions of the respiratory tract of CF patients and, therefore, persists in spite of antibiotic therapy.

## Results

### CIP exposure selects for CIP-resistant variants with prolonged lag phase

Growth curves of 92 CIP-resistant isolates (47 WT PAO1 and 45 Δ*katA*) representing 57 isolates recovered from CIP-evolved populations and 35 isolates recovered from CTRL-evolved populations were analyzed.

Three parameters of bacterial growth were calculated: maximum growth density, doubling time, and time to initiation of the exponential phase (lag time).

**Fig. 2:**
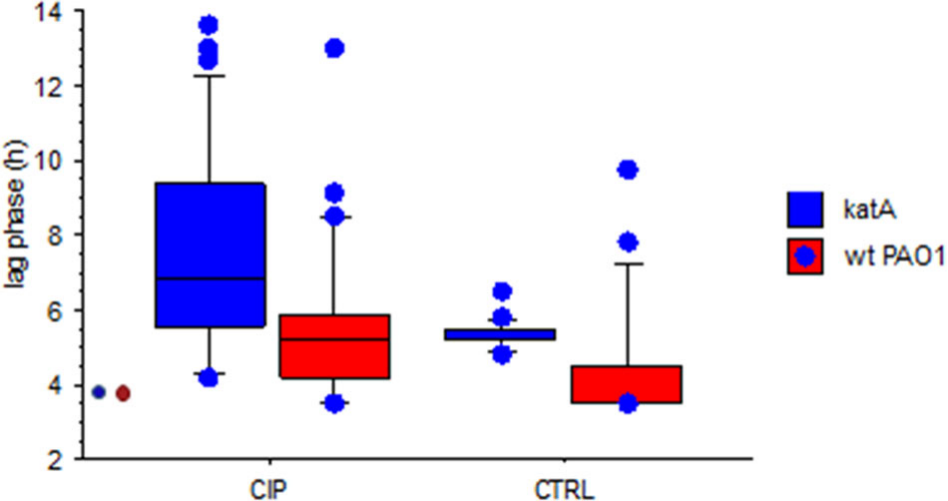
Box and whisker plots representing the median, 25, 75, and 90 centiles of the lag time (h) of CIP-resistant colonies isolated from evolution under CIP exposure (CIP) and without antibiotic exposure (CTRL). **a** Significant longer lag phase was observed in CIP-evolved compared to CTRL-evolved PAO1 colonies (in red) (*p* = 0.002) and Δ*katA* (in blue) (*p* = 0.002). The lag phase of the ancestor colonies is represented by the red (PAO1) and the blue (Δ*katA*) dot. Mann–Whitney test for nonparametric data was used to compare the different groups.

CIP-resistant colonies from CIP-evolution had significantly extended lag phase compared to CTRL (*p* = 0.002). For WT PAO1, the lag phase (median (range) in h) was 5.36 (3.5–13) vs. 3.5 (3.5–9.7) while for Δ*katA* it was 6.84 (4.2–14) vs. 5.2 (4.8–6.5) (Fig. 2). The extended lag phase was more frequent in Δ*katA* colonies compared to WT PAO1, and it occurred also in some of the CTRL colonies suggesting adaptation to growth in media during the evolution experiment.

For CIP-resistant colonies of the WT PAO1, a statistically significant (*p* = 0.0007) increase in the doubling time was found for the colonies isolates from CIP evolved compared to CTRL populations. The doubling time (median (range) in min) was 97.5 (71–893) vs. 69 (69–104). This was not observed for colonies isolated from the planktonic evolution experiment

### The fitness cost of CIP-resistance development

To determine the fitness cost of the CIP resistance, the fitness index (FI) of 42 CIP-resistant colonies (24 colonies from CIP-evolved populations and 18 colonies from CTRL-evolved populations) (Supplementary Table 1) were investigated in competition experiments started at ratio 1:1 between the evolved CIP-resistant and the ancestor colonies in both planktonic and colony-biofilm cultures. The competition in colony-biofilm cultures was conducted in the presence and absence of 0.1 mg/L CIP.

The FI was expressed as the ratio between the doubling- time of the CIP-resistant colony and the ancestor colony after 24 h incubation (see Supplementary methods).

In planktonic competition studies, the CIP-resistant colonies showed decreased fitness compared to the WT ancestors (FI < 1), with the exception of CIP_Pl_10, CIP_Pl_11, and CIP_Pl_14. The highest fitness cost was observed for the CIP-resistant colonies isolated from the planktonic evolution of Δ*katA* and the lowest for CIP-resistant colonies isolated from planktonic evolution of PAO1.

**Fig. 3:**
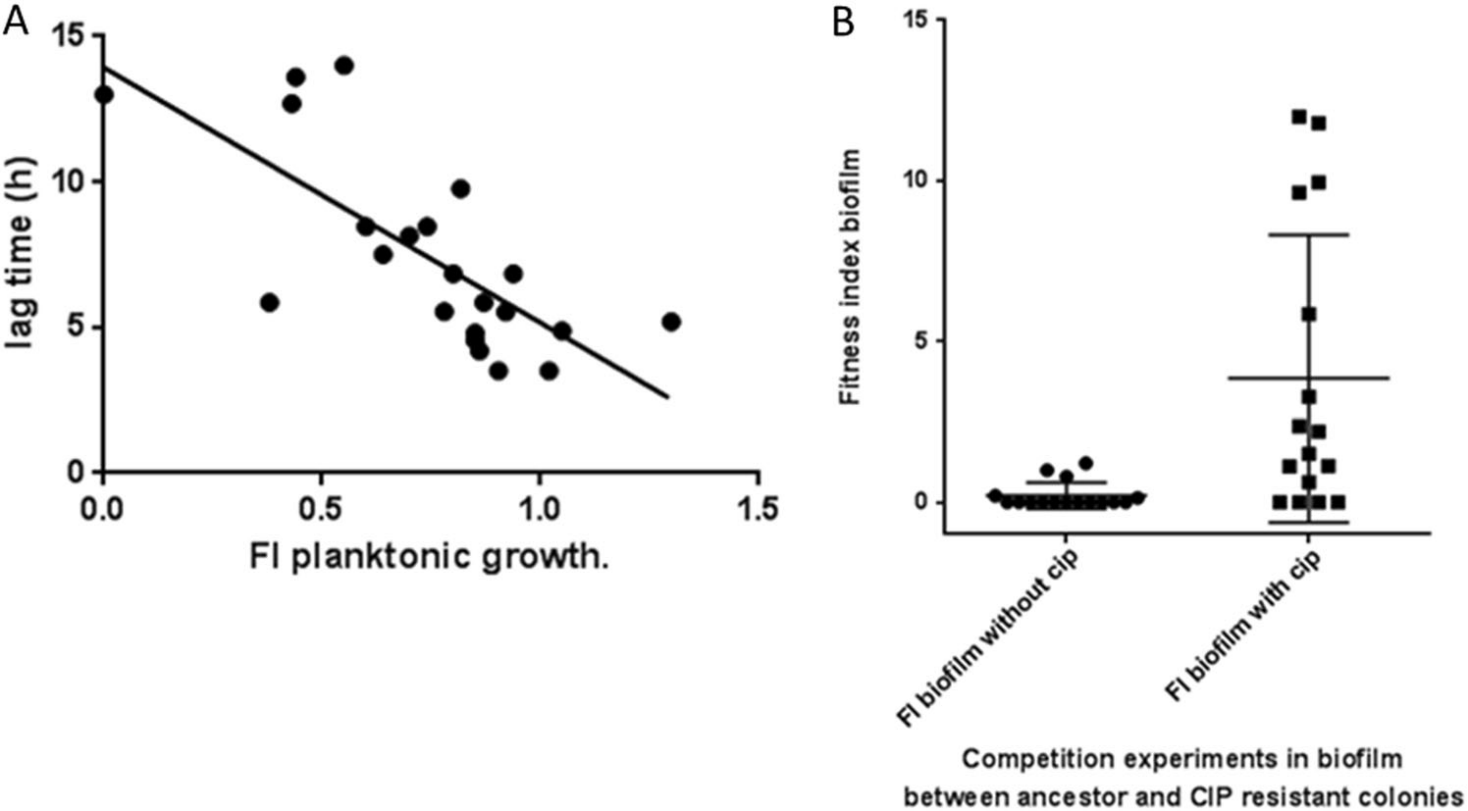
Relative fitness (Fitness index) of the evolved CIP-resistant isolates compared to the ancestor colonies. **a** An Inverse correlation (*R*^2^ = 0.53) between the lag time and the planktonic fitness index (FI) was observed. **b** CIP-resistant colonies had a significant (*p* = 0.0005) better biofilm FI in CIP-exposed biofilms compared to unexposed. Wilcoxon matched-pairs signed ranks was used as statistical test.

An inverse correlation (*R*^2^ = 0.53) between the lag time and the planktonic FI was observed: the shorter the lag time, the better the fitness (Fig. 3a).

The FI in a 24 h colony-biofilm without exposure or with 24 h exposure to 0.1 mg/L CIP are shown in Supplementary Table 2. In colony-biofilm competition studies, few CIP-resistant isolates were identified in the mixed 48 h biofilm population. However, this was dramatically changed when a 24 h-old biofilm was exposed to 0.1 mg/L ciprofloxacin for 24 h. In the latter case, the CIP-resistant colonies had a significant (*p* = 0.0005), growth advantage compared to ancestor colonies (Fig. 3b) (Supplementary Table 2).

### CIP exposure selects for CIP-resistant colonies with impaired twitching but maintained swarming motility

**Fig. 4:**
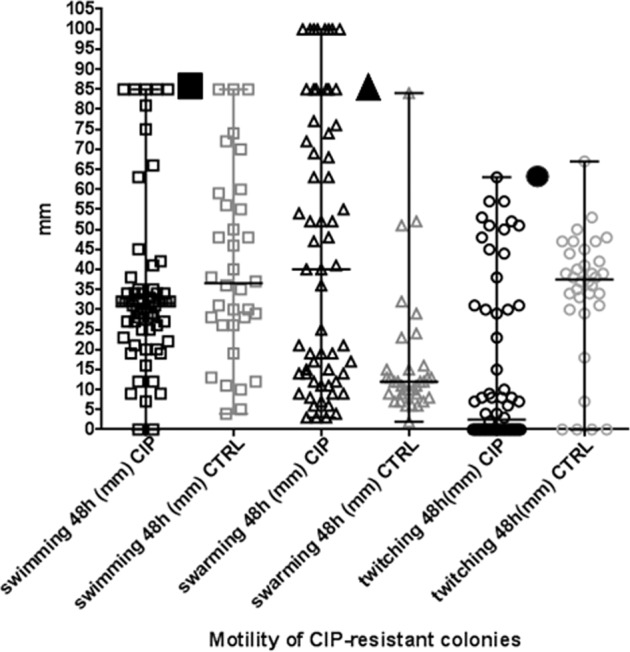
Motility of CIP-resistant colonies Plots representing the swimming (squares), swarming (triangles) and twitching (dots) motilities (median, and the ranges) in (mm) of CIP-resistant colonies isolated from evolution experiments under exposure to CIP (CIP) or without CIP exposure (CTRL). For comparisons, the motilities of the WT PAO1 are presented by black symbols in the figure. A significant lower twitching motility (*p* = 0.03) and higher swarming motility (*p* = 0.0001) was observed in CIP-resistant colonies isolated from CIP compared to and CTRL biofilms. Mann–Whitney test was used for statistical analysis.

The swimming, swarming and twitching motility of 92 CIP-resistant isolates were analyzed (57 CIP and 35 CTRL) (Fig. 4).

No significant difference in swimming motility between the CIP and CTRL isolates was observed, though for both groups the CIP-resistant isolates were impaired in swimming compared to ancestor colonies.

CIP-resistant colonies from CTRL-evolved populations had significantly lower swarming motility (*p* = 0.0001) and higher twitching motility (*p* = 0.03) compared to CIP-evolved populations.

### Cross-resistance to beta-lactam antibiotics

**Fig. 5:**
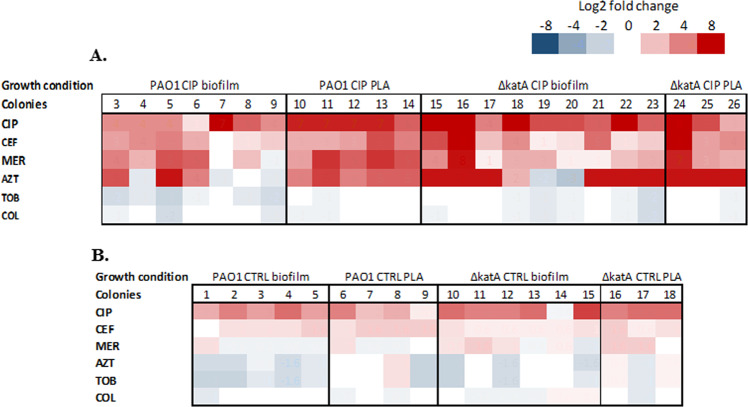
Cross-resistance and collateral sensitivity to different antibiotics. Heat map showing the changes in the resistance levels to six classes of antibiotics. Results shown are the log2 fold change in the minimum inhibitory concentrations (MIC) (mg/L) in the evolved CIP-resistant isolates compared to the sensitive ancestral strain. **a** The relative changes in the MIC levels in CIP-resistant isolates recovered from CIP-evolved populations. **b** The relative changes in the MIC levels in CIP-resistant isolates recovered from CTRL-evolved populations. CIP ciprofloxacin, CEF ceftazidime, MER meropenem, AZT aztreonam, TOB tobramycin, COL colistin.

The MIC of ciprofloxacin, ceftazidime, aztreonam, meropenem, tobramycin, and colistin in CIP-resistant (24 colonies from CIP-evolved populations and 18 colonies from CTRL-evolved populations) were determined and are presented in Fig. 5a, b and Supplementary Table 3.

The level of resistance was higher in the CIP-resistant colonies from CIP-exposed populations compared to CTRL population and simultaneous resistance to beta-lactams such as aztreonam and ceftazidime was observed, in many cases with MICs above the clinically resistance breakpoints (Supplementary Table 2). However, CIP-resistant isolates showed decreased MICs to tobramycin and colistin antibiotics.

No correlations between the CIP MIC levels and the fitness index, lag period or doubling time were observed.

Growth of resistant subpopulations in the inhibition zone of the antibiotic strip were observed for all the CIP-resistant colonies with hypermutable phenotype (Supplementary Table 3) but also in other populations of single CIP-resistant colonies suggesting a hetero-resistant phenotype, especially to meropenem.

### Genomic mutations of the evolved-resistant isolates that could explain the observed phenotypes

To elucidate the genetic mechanisms underlying the antibiotic resistance and the evolved phenotypes in the evolved CIP-resistant colonies, we have sequenced 24 CIP-resistant colonies recovered from CIP-evolved populations (16 colonies isolated from biofilm populations and 8 colonies isolated from planktonic populations) and 18 CIP-resistant colonies recovered from CTRL-evolved populations (11 colonies isolated from biofilm populations and 7 colonies isolated from planktonic populations) for both WT PAO1 and *ΔkatA*. The genomic DNA of original ancestors PAO1 and *ΔkatA* were sequenced as well. The identification and phenotypes of the sequenced colonies are presented in Supplementary Tables 1 and 2a, b.

Mutations with frequencies above 80% were prioritized for determining the number of mutations and calculating the ratio between nonsynonymous and synonymous mutations d*N*/d*S* (Table 1). In CIP-evolved populations the ratio d*N*/d*S* is higher than in CTRL populations suggesting a positive selection. To get more insights into the mutational landscape, we also considered mutations with frequencies below 80% and higher than 10%. (Supplementary Data Set).

**Table 1:** The types of mutations (number and % of total number of mutations) in CIP and CTRL-resistant colonies in WT PAO1 and *ΔkatA* biofilm and planktonic (PL) *P. aeruginosa*.

	Transitions	Transversions	Insertions/deletions	Inversions/duplications	Total number of mutations	d*N*/d*S*
CIP-resistant colonies						
PAO1_biofilm	6 (24%)	3 (12%)	13 (52%)	3 (12%)	25	2.7
PAO1_PL	5 (18%)	6 (21%)	13 (46%)	4 (14%)	28	1.6
*ΔkatA*_biofilm	157 (78%)	3 (1.5%)	43 (21%)	3 (1%)	206	1.5
*ΔkatA*_PL	1 (8%)	3 (25%)	8 (89%)	0	12	0.9
CTRL-resistant colonies						
PAO1_biofilm	1 (8%)	5 (42%)	6 (50%)	0	12	2
PAO1_PL	3 (18%)	0	7 (41%)	7 (41%)	17	1
*ΔkatA*_biofilm	1 (6%)	1 (6%)	14 (82%)	1 (6%)	17	0.1
*ΔkatA*_PL	3 (38%)	0	5 (63%)	0	8	0.4

The ratio between the nonsynonymous/synonymous mutations (d*N*/d*S*) is presented.

**Fig. 6:**
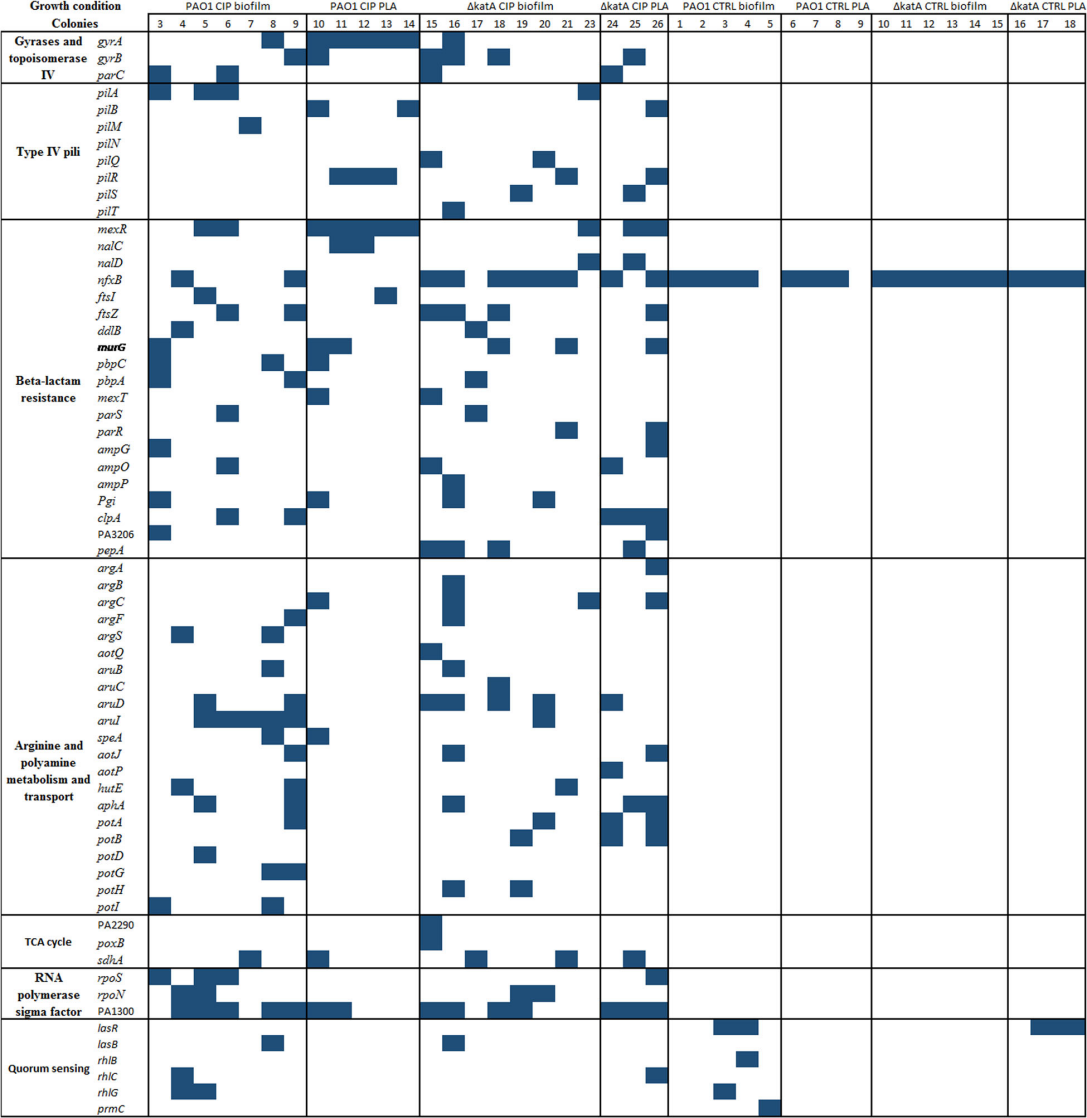
A distribution map of the mutated genes belonging to different functional categories in the CIP-resistant colonies isolated under different conditions. The blue boxes indicate the genes with nonsynonymous mutations identified in the individual CIP-resistant colonies recovered from CIP- and CTRL-evolved biofilm and planktonic (PLA) populations. The identity number of the colonies is shown in Supplementary Table 1.

Gene mutations which are repeatedly observed after independent exposures to a condition provide strong evidence for adaptive evolution. To identify common genes that were mutated under evolution at sub-MIC CIP, we focused on genes which were recurrently mutated in several of the CIP-resistant colonies^16^. A map showing the presence or absence of the mutated genes in different functional classes related to the observed phenotypic shifts is presented in Fig. 6.

### Mutations in CIP-resistant colonies from CIP-evolved populations

#### Mutations in antibiotic resistance determinants

Common evolutionary trajectories among CIP-resistant colonies leading to resistance to CIP were observed.

In CIP-resistant colonies from WT PAO1 planktonic populations, simultaneous mutations in gyrase *gyrA* and efflux-pump regulators of MexAB-OprM *(mexR*, *nalC*, and *nalD*) at high frequencies might explain the high-MIC values to ciprofloxacin. In addition, in one of the colonies CIP_Pl_10, mutations in PA3228 gene that encodes for a putative ATP-binding/permease fusion ABC transporter that may function as a multidrug transporter was observed with high frequencies (>80%).

In the CIP-resistant colonies from WT PAO1 biofilm populations, *nfxB* gene, a negative regulator of efflux-pump MexCD-OprJ was repeatedly mutated as well as other efflux-pump regulator genes, such as *mexR* and *mexS*, at high frequencies. The MIC ciprofloxacin levels in these colonies are lower than in planktonic colonies with the exception of CIP_BF_5 (Supplementary Tables 1, 3) with high MIC of CIP of 32 mg/L in which an additional mutation in PA3228 gene encoding an ABC transporter was observed at low frequencies.

In CIP-resistant colonies from *ΔkatA* planktonic populations, simultaneous mutations in *mexR* or *nalD* at high frequencies and *parC* at low frequencies can explain the CIP-resistant phenotype. In one colony CIP_Pl_25 (Supplementary Tables 1, 3) with a subpopulation with MIC to ciprofloxacin of 6 mg/L, an additional mutation in PA3228 was also identified at low frequencies.

In CIP-resistant colonies of *ΔkatA* biofilm populations, three colonies (CIP_BF_15, CIP_BF_16, and CIP_BF_18) had a 28 nucleotide insertion in *mutL* causing a hypermutable phenotype (Supplementary Data Set). In the hypermutable colonies simultaneous mutations in gyrase *gyrB, parC*, as well as efflux-pump regulators such as *nfxB* at high frequencies might explain the high-MIC values to ciprofloxacin. In one of the colonies CIP_BF_15, mutations in the ABC transporter PA3228 gene were found at high frequencies (>80%).

A list of mutated genes that might explain for the observed cross-resistance of CIP-resistant colonies to beta-lactams, and especially aztreonam is presented in Fig. 6.

In CIP-resistant colonies isolated from evolution under CIP exposure, mutations in efflux pumps regulators (*nfxB*, *nalC*, *nalD*, and *mexR*) were identified. These mutations cause upregulation of the MexCD-OprJ and MexAB-OprM pumps which, besides ciprofloxacin, also accommodate beta-lactams. Interestingly, in addition we detected mutations in genes encoding for proteins involved in cell division (the divisome) (for example, *ftsI*, *ftsZ*, and *murG*), penicillin-binding protein *pbpAC* and *amp* genes involved in the regulation of AmpC beta-lactamase (Fig. 6).

#### Mutations in genes of metabolic pathways

In CIP-resistant colonies there were mutations at frequencies higher than 80% in different genes related to the arginine metabolism and transport such as *argS* and *aotQ* in colonies CIP_BF_4, CIP_BF_8 of the WT PAO1 and CIP_BF_15 of Δ*katA*. While at frequencies lower than 80%, mutations in *aruB*, *aruD, aruI*, *speA*, *argC*, *aotJ* and *hutE* were repeatedly detected in CIP-resistant colonies of the WT PAO1 and *ΔkatA* CIP-resistant colonies (Fig. 6).

We have also detected mutations in genes related to the metabolism and transport of polyamines, as arginine is one of the precursors for polyamine synthesis. Mutated genes, such as *aphA*, *potA*, and *potB* were detected in several colonies (Fig. 6).

In CIP-resistant colonies isolated from biofilm, mutations in PA1300 encoding for σ factor 70 and in *rpoN* encoding for σ factor 54 were observed repeatedly and this might correlate to the observed prolonged lag phase observed in these colonies.

In addition, mutations in *rpoS* gene that codes for stress response regulator RpoS were detected in five CIP-resistant colonies (colonies no. CIP_BF_3, CIP_BF_5, CIP_BF_6, and CIP_Pl_26). It is noteworthy that these mutations are not present in WT CTRL-resistant colonies. These mutations might explain the increased doubling time of the CIP-resistant colonies.

In some of the CIP-resistant colonies, mutations in genes related to TCA cycle were observed. In colonies no. CIP_BF_7, CIP_BF_21, and CIP_Pl_25, the *sdhA* gene that encodes succinate dehydrogenase was mutated at frequencies higher than 80%. In addition, *poxB* and PA2290 genes that encodes pyruvate and glucose dehydrogenases were also found to be mutated.

In the *ΔkatA*-hypermutable CIP-resistant colonies, the genes related to iron storage and transport were mutated such as *fecA*, *fecI*, *hasR*, and PA2911 that encodes *tonB* dependent receptors at frequencies above 80% (Supplementary Data Set).

#### Mutations in CIP-resistant colonies from CTRL-evolved populations

Surprisingly, in CIP-resistant colonies from CTRL populations, *nfxB* gene was also frequently mutated in the majority of CTRL-resistant colonies showing that population with decreased susceptibility to CIP can arise in biofilm and planktonic growth modes without exposure to antibiotics.

Quorum-sensing genes such as *lasR* and *prmC* were mutated in CTRL –resistant colonies of both WT PAO1 and *ΔkatA* in colonies CTRL_BF_3, CTRL_BF_4, CTRL_BF_5, CTRL_Pl_17, and CTRL_Pl_18. This might explain the impaired swarming motility observed in CTRL CIP-resistant colonies compared to CIP-exposed colonies.

#### Mutations in genes considered patho-adaptive in chronic infections

**Fig. 7:**
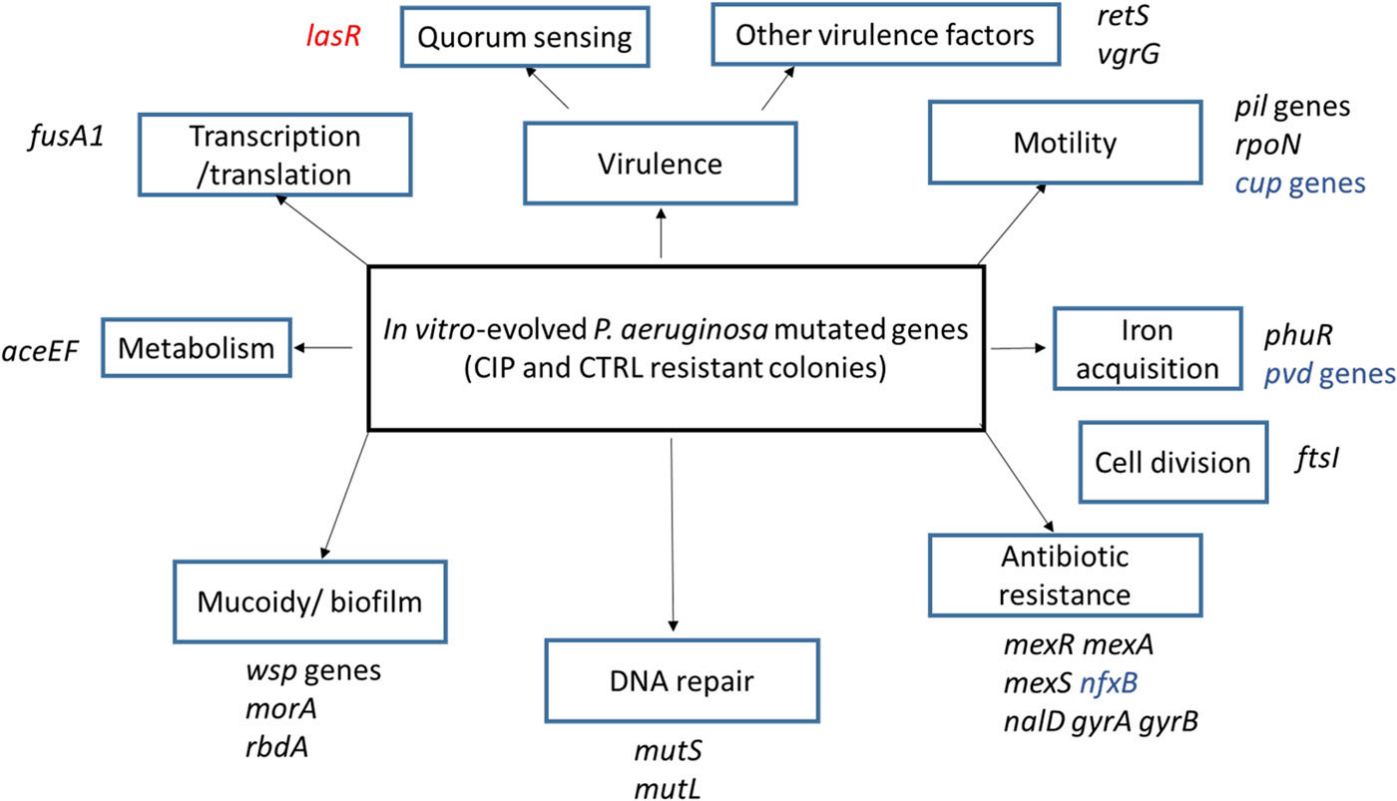
Common genes mutated in evolved *P. aeruginosa* isolates under in vivo and in vitro conditions. This diagram shows the mutated genes in the evolved-resistant isolates recovered from the in vitro experimental evolution of *P. aeruginosa* that are correlated with the patho-adaptive genes detected in naturally evolved clinical isolates inside the CF lungs^18,33,39,43^. The genes that are only mutated in CIP-evolved colonies are in black, those mutated only in CTRL-evolved colonies are highlighted in red while the genes that are mutated in both CTRL- and CIP-evolved colonies are highlighted in blue. The threshold of the frequency of the mutated genes in evolved isolates is higher than 10%.

We queried if the mutated genes identified during in vitro experimental evolution were of relevance for the in vivo evolution, and we looked for common genes with the patho-adaptive genes found to be mutated in several *P. aeruginosa* isolates from chronic lung infections of patients with CF^17,18^. A list of common genes encountered during in vivo and in vitro evolution is presented in Supplementary Table 4 and Fig. 7.

## Discussion

Extensive phenotypic and genotypic changes compared to the ancestor colonies have been observed in the CIP-resistant colonies isolated from *P. aeruginosa* WT and Δ*katA* biofilm and planktonic evolution experiments in the presence of sub-MIC concentration of CIP. The evolution in the presence of subinhibitory concentrations of CIP selected for CIP-resistant mutants with distinct phenotypes compared to those isolated from CTRL populations.

We show that pathways of CIP-resistance development depend on of the bacterial life-style. High-level CIP-resistant mutants were isolated especially from evolution in planktonic populations while low-level CIP-resistant mutants were isolated from evolution in biofilm. The genetic basis for the two distinct phenotypes were simultaneous mutations in quinolone resistance determinants *gyrA*, *parC*, and efflux-pump regulators in high CIP-resistant mutants from planktonic populations as repeatedly published^9,14,15,19^ and mutations in efflux-pump regulators for CIP-resistant mutants evolved in biofilms. Similar observations have been recently published in *Acinetobacter baumanii*^20^ suggesting that this is a general characteristic of evolution in biofilms.

Low-level CIP-resistant mutants due to mutations in *mexZ*, a negative regulator of MexXY-OprM efflux-pump associated to active efflux of aminoglycosides and ciprofloxacin were reported in CF *P. aeruginosa* isolates from the initial phase of the chronic lung infection, suggesting that this may contribute to in vivo persistence^21^.

Association between resistance to CIP and beta-lactams was observed in this study (Supplementary Table 3). Here, we detected many genes especially involved with cell division and TCA cycle responsible for high resistance to ceftazidime and aztreonam antibiotics, confirming previous findings^22,23^ (Figs 5 and 6). Cross-resistance between CIP and beta-lactam antibiotics was also found in the planktonic experimental evolution study of *P. aeruginosa*^9^. In addition, our data show that CIP-resistant colonies displayed collateral increased sensitivity to both tobramycin and colistin. Previous studies^24^ showed that CIP-resistant isolates of *P. aeruginosa* are susceptible to aminoglycosides. This might be explained by the mutations in *nfxB* which has been shown to correlate to decreased activity of the MexXY-OprM and collateral sensitivity to aminoglycosides^25^. However, the minimal increase in the sensitivity to aminoglycosides and colistin of *nfxB* mutants has probably no or limited impact on the clinical efficacy of the treatment with these antibiotics since clinical treatments use concentrations that are far above these MICs. An extended lag phase and a reduction in max growth in LB were observed in CIP-evolved colonies. This is in accordance to recent studies investigating the metabolic functionality of experimentally evolved antibiotic-resistant *P. aeruginosa* which showed that CIP-evolved lineages exhibited longer lag phases and longer doubling times^26^.

The importance of the lag phase in the bacterial response to antibiotics has been previously shown by Fridman et al.^27^, who reported that “tolerance by lag” allows bacteria to survive under high antibiotic concentrations, and may facilitate the subsequent development of resistance. However, in our studies, the extension of the lag phase is not an adaptive response to antibiotics as the growth curves of the CIP-resistant colonies were conducted in the absence of the antibiotic, and the CIP-resistant colonies have been passed twice in antibiotic-free media after isolation from the population analysis plates. The mutations identified in the different metabolic pathways and RNA polymerase sigma factors might explain this phenotype.

Competition experiments in planktonic cultures showed that CIP resistance was associated with a fitness cost. However, in biofilm competition studies the resistant colonies persisted as a very small subpopulation and overgrew the ancestor colony in the presence of 0.1 mg/L CIP. Persistence of CIP-resistant colonies in biofilms in spite of their fitness cost suggest that growth rates are less important for survival in this mode of growth which probably allows maintenance of resistance for longer period of time in the absence of selective pressure.

Swarming, which is QS regulated^28,29^, was maintained in CIP-resistant colonies evolved under selection with sub-MIC CIP while CTRL CIP-resistant colonies lost the swarming. The mutations in the QS genes found in CTRL CIP-resistant colonies can explain this phenotype. This is in accordance with a recent published study confirming the lack of selection of QS mutants (*lasR*) during evolution experiments in the presence of antibiotics^30^.We have previously shown in planktonic experimental evolution studies that higher levels of QS molecules in *P. aeruginosa* populations evolved under sub-MIC ciprofloxacin compared to CTRL^10^.

It has been shown that swarming *P. aeruginosa* exerts adaptive resistance to a number of antibiotics, including CIP, which might explain the maintenance of the swarming motility during evolution in the presence of CIP^28^.

The loss of twitching motility is correlated to the frequent mutations in *pil* genes encoding for type IV pili identified in CIP-resistant colonies. The association between CIP resistance and mutations in *pil* genes has been reported by other groups but the reason for this association is not completely clear^24^.

Mutations in genes responsible for the catabolism of arginine such as *aruL* involved in the arginine decarboxylase and *arcA* involved in the arginine deaminase catabolic pathways were identified. Impairment of these pathways promotes the utilization of arginine by nitric oxide synthase to convert it to nitric oxide^31,32^. Nitric oxide will promote anaerobic respiration, and this is in agreement with previous studies that showed that anaerobic respiration is promoted in *P. aeruginosa* under CIP treatment^10,33^. Nitric oxide has been shown to be involved in the biofilm disruption^34^, but if this would occur as a consequence of the metabolic rewiring in CIP-treated biofilms remains to be investigated.

Antibiotic-resistant *P. aeruginosa* mutants overexpressing efflux pumps were shown to rewire their metabolism to avoid fitness cost including increased expression of the anaerobic nitrate respiratory chain when cells are growing under aerobic conditions^35^.

In accordance with the metabolic rewiring in the presence of ciprofloxacin, we have detected mutations in the enzymes involved in the TCA cycle in CIP-resistant colonies. This is in agreement with studies^5^ showing that mutants with impaired activity of TCA cycle enzymes were more tolerant to antibiotics due to decreased levels of bactericidal hydroxyl-radicals^36^.

Interestingly, mutations in the enzymes of the TCA cycle have been reported in clinical *P. aeruginosa* isolates that have evolved in the lung of CF patients^37^ and associated to the impaired catabolic capacity of the isolates. We have previously reported that metabolic reduction is a common adaptive trait of the *P. aeruginosa* nonmucoid isolates in the CF lung^38^ which has probably an impact on the persistence of the bacteria in the lung, in spite of the antibiotic treatment.

In addition, RNA polymerase *σ*^54^ (*rpoN*) and *σ*^S^*(rpoS)* were found to be mutated in some CIP-biofilm resistant colonies and Δ*katA* planktonic-resistant colonies and associated to impaired growth of the isolates. However, mutations in these genes were not detected in WT PAO1 planktonic-CIP and either of the CTRL-resistant colonies. Interestingly, *rpoN* mutants were found in *P. aeruginosa* from chronically infected patients^17,39^.

A comparison of the identified mutated genes in CIP-resistant colonies from in vitro-evolved populations with a list of patho-adaptive genes found to be mutated in several *P. aeruginosa* isolates from chronic lung infections of patients with CF^17,18^ revealed a large degree of overlap (Fig. 7; Supplementary Table 4) suggesting the important role played by antibiotic exposure of biofilm-growing bacteria in shaping the adaptation in vivo. This is in agreement with previous published results showing that, in vitro evolution can be used to predict in vivo adaptive changes in the presence of antibiotics^24^. However, some patho-adaptive genes, for example those involved in conversion to mucoidity and biofilm formation (*mucA*, *algU*, *algG*, *pelA*, and *rbd A*) were only mutated in clinical isolates suggesting the role of the immune system in the selection of these phenotypes, in accordance to our previous publication^40^.

In the future, supplementation of the present results with gene expression data will improve the understanding of evolution processes in biofilms.

In conclusion, our data on the phenotypic and mutagenic evolution in *P. aeruginosa* biofilms support the mutagenic potential of CIP at subinhibitory concentrations^41^. Mutations in CIP-resistance determinants were associated with mutations conferring phenotypes supporting a general survival advantage in the presence of antibiotics, as they were affecting some of the common mechanisms of action described for antibiotics, such as switch of the metabolic pathway to anaerobic respiration or affecting the TCA cycle. Phenotypes such as increased lag phases will also impair the effect of other antibacterial agents, while loss of twitching might impair biofilm dispersal^42^. Overall, our data show that CIP-resistant mutants acquire several other mutations that might confer survival advantages during antibiotic treatment of biofilms.

## Methods

### Strains

The wild-type (WT) *P. aeruginosa* PAO1 and its catalase mutant (Δ*katA*) (MIC CIP = 0.125 µg/ml) have been used previously to start biofilm and planktonic evolution experiments in the presence of 0.1 mg/L ciprofloxacin (CIP) or without selection pressure (CTRL) for seven passages, as previously published^14,15^. The evolved populations were plated on LB plates containing ciprofloxacin (0.5, 1, and 2 mg/L) (population analysis) and three colonies from the plates with the highest ciprofloxacin concentration which allowed growth were isolated, passed twice on antibiotic-free media and stored at −80 ^o^C for further analysis (see Fig. 1a). CIP-resistant colonies isolated after the last passage of the biofilm and planktonic evolution experiments were used for further investigations in this study.

For growth curves and motility assays all the available CIP-resistant colonies from CIP and CTRL evolution experiments, which are in total 92 isolates, were investigated.

For whole-genome sequencing, susceptibility testing to antipseudomonal antibiotics and fitness cost determinations in both planktonic and biofilm growth, a total of 42 colonies were investigated; 24 colonies from CIP-evolved populations (16 colonies isolated from biofilm populations and 8 colonies isolated from planktonic populations) and 18 colonies from CTRL-evolved populations (11 colonies isolated from biofilm populations and 7 colonies isolated from planktonic populations). The selection was randomly done and aimed at including colonies representing the different replicate lineages (Supplementary Table 1). Four of the Δ*katA* CIP-resistant colonies had hypermutable phenotype, as indicated in Supplementary Table 1.

### Phenotypic characterizations

Growth curves, MIC determinations, fitness cost determination in planktonic and biofilm cultures and motilities (swimming, swarming, and twitching) were performed, as described in Supplementary methods.

### WGS and analysis

The whole-genome sequencing was performed for 42 CIP-resistant colonies (24 isolated from CIP-evolved population and 18 from CTRL-evolved populations) as well as for WT PAO1 and Δ*katA* ancestor colonies. Genomic DNA was extracted from the colonies using Gentra puregene yeast/bacteria DNA purification kit (Qiagen). The DNA was prepared for sequencing using the Illumina TruSeq DNA Nano kit and sequenced on an Illumina MiSeq yielding a coverage of approximately 120×. Sequencing reads were mapped to the reference genome of *P. aeruginosa* PAO1 (GenBank accession. NC_002516), and single and multiple nucleotide variants (SNVs and MNVs) were called using CLC genomic workbench (Qiagen). Large indels and structural variants were also detected using using CLC genomic workbench. Mutations present in the CIP-resistant colonies were filtered out from the genome of the sequenced WT PAO1 and Δ*katA* ancestor colonies using customized Linux and AWK scripts. R (version 3.2.5) was used for further statistical analysis of the mutations detected in colonies and all mutations occurring in >10% of the reads and at least 10 unique reads were included in the analysis.

The Pseudomonas Genome Database was used for gene function analysis. d*N*/d*S*, the ratio of the rate of nonsynonymous substitutions (d*N*) to the rate of the synonymous substitutions (d*S*), was calculated as a measure of the selection pressure acting on the protein-coding genome, as previously described, assuming that 25% of all single-nucleotide polymorphisms (SNP) result in synonymous changes^33^. d*N*/d*S* is expected to be >1 if natural selection promotes changes in protein sequences and <1 if natural selection suppresses changes.

### Statistical analysis

Graphs and statistical analysis were performed using GraphPad Prism 7, Statview software and R (version 3.2.5). Student t-test and Mann–Whitney for nonparametric were used for comparisons among resistant isolates for growth rates and motilities (comparing CIP populations to CTRL populations and CIP-biofilm populations to CIP planktonic populations). The differences were considered significant when the *p* value was ≤0.05.

### Reporting summary

Further information on experimental design is available in the Nature Research Reporting Summary linked to this article.

## Supplementary information

Supplementary Methods

Supplementary Data

Reporting Summary

## Data Availability

The datasets generated or analyzed during this study are available and included in this publication (and its published supplementary files). The project information is accessible with the following link http://www.ncbi.nlm.nih.gov/bioproject/643668.
