# Supplementary material for: The evolutionary trajectories of P. aeruginosa in biofilm and planktonic growth modes exposed to ciprofloxacin: beyond selection of antibiotic resistance
Source: NPJ Biofilms Microbiomes. 2020 Jul 24;6:28. doi: 10.1038/s41522-020-00138-8 (PMC7381665; doi:10.1038/s41522-020-00138-8)
Supplement: Supplementary file 1 — Supplementary Methods [file 41522_2020_138_MOESM1_ESM.pdf]

Supplementary methods

Methods employed for phenotypic characterization

Growth curves

Overnight cultures in LB of the CIP resistant colonies were diluted 10^{-4} and 100µl were transferred in triplicates in microtiter plates which were incubated in Infinite F200 Pro plate reader (Tecan) with lid on, at 37°C and shaken at 225 rpm for 24 h. Absorbance (OD600nm) was measured using Magellan software, every 20 minutes during 24h incubation. Growth curves were constructed and used to calculate the lag-phase, the doubling time and max OD.

Fitness-cost determination of CIP resistant isolates in competition studies

P. aeruginosa PAO1 and Δ*katA* ancestor strains were chromosomally tagged with green-fluorescent protein (GFP) by the Tn7 system with streptomycin (Strep.) resistance marker and were employed for planktonic and biofilm competition studies between the ancestors and the evolved CIP resistant colonies. GFP tagging did not affect the growth rates of *P. aeruginosa* PAO1 and Δ*katA.*Details of the competition experiments are described in supplementary material (suppl.material)

Competition in planktonic and biofilm cultures

Competition in planktonic cultures

Overnight cultures in LB of the ancestor colonies (GFP tagged *P. aeruginosa* PAO1 or Δ*katA*) and of the evolved CIP resistant colonies were diluted to OD=0.01 in LB and were mixed 1:1 (ancestor/resistant colony) and incubated at 37 ^{o}C with shaking 180 rpm.

Samples from the mixture of the two strains were taken at 30 min and 7 h or 24 h (for slow grower) and the CFU was counted on LB plates and on LB plates supplemented with 100 mg/L Strep. The LB + 100mg/L Strep plates are selective for the tagged ancestor strains. The CFU of the CIP resistant colonies was calculated by extracting the CFU counts on LB+ 100 mg/L Strep from the CFU counts on LB plates. In addition, to verify the number of ancestor cells on the LB plates, GFP of the tagged ancestor colonies was observed under an epifluorescence microscope (Leitz Aristoplan) with blue excitation light (488 nm, fluoresceine isothiocyanate (FITC) filter) and the GFP positive colonies were counted.

CFU counts were used to calculate the doubling time of each strain and the fitness index in planktonic growth was defined as the ratio between the resistant and ancestor colony.

Competition in Colony-Biofilm

Overnight cultures were diluted to OD=0.05 and were mixed 1:1 (ancestor/resistant colony) (1ml + 1ml). Five µl of the mixture diluted 1:10 were spot-inoculated on the top of polycarbonate membrane filters (Whatman Nuclepore membranes, 25-mm diameter, 0.2-µm pore size). Two membranes were placed on LB plates and incubated at 37 ^{o}C for 24 h.

The sizes of each bacterial population in the initial inoculum (ancestor and resistant colony) were measured by plating the mixture on LB and LB with 100 µg/ml Strep.

One of the two membranes with 24 h colony-biofilm was transferred on LB plate and one on LB + 0.1 mg/L CIP (the concentration that was used in the evolution experiment) and incubated further for another 24h.

After 24 h, the colony-biofilms were harvested, sonicated and plated on LB and LB + 100 mg/L Strep, as previously described.

The doubling time of the ancestor and resistant colonies in biofilms in the presence and absence of CIP were calculated and the fitness index in biofilm was defined as the ratio between the doubling time of the resistant colony and the ancestor in biofilm in the absence or presence of CIP.

w is fitness, *A* and *B* are the population sizes of the two competitors, subscripts *i* and *f* indicate the initial and final time points in the assay; here, ln refers to the natural logarithm in order to reflect population growth, although the ratio used to express fitness is insensitive to the choice of base used.

$$w = \frac{\ln\left(\frac{A_f}{A_i}\right)}{\ln\left(\frac{B_f}{B_i}\right)}$$

Motility

Swimming

Cells from one colony were inoculated with an inoculating loop on trypton agar swimming plates (1% tryptone, 0.5% NaCl, 0.3% agar) and incubated for 24 and 48h at 30°C. The area of the bacterial swimming from the inoculated spot was measured (10).

Swarming

The colonies were grown overnight in LB and 2 µl of the overnight culture was spotted at the center of a swarming plate and the spot was left 20-30 min to dry before incubation for 24 and 48h at 30°C.The area of swarming was measured. (swarming plate: minimal media: 500µl $MgCl_2$ (1M), 500 µl CaCl2 (0.1M), 2.5g Bacto-agar, 426.5 ml milliQ H_2O, 50 ml A10, 10 ml glucose (10%) and 12.5 ml casamino acids (20%) to 520C, mix everything together and poor plates).

Twitching

Cells from one bacterial colony were inoculated with an inoculating loop in 1%LB agar at the bottom of the agar plate. The plates were incubated at 37°C for 24 and 48h and the area of twitching was measured.

MIC determinations

The susceptibility to ciprofloxacin, ceftazidime, aztreonam, meropenem, tobramycin and colistin of CIP-resistant colonies isolated from the population analysis of the evolved biofilm or planktonic populations were performed by e-test, as recommended by the manufacturer.

Supplementary table 1. Identification of the 42 CIP resistant colonies that have been sequenced. The colonies are isolated from CIP and CTRL evolved biofilm (BF) and planktonic (PL) populations of WT PAO1 and Δ*katA*. A6, B6, C6 and D6 represent the lineage name and passage number 6 from which these colonies are isolated. I, II, III represent the number of evolution experiment and the numbers at the end represent the number of the colony as there were three colonies isolated per each population. Hp indicates that the colony is isolated from a hypermutable lineage.

CIP resistant Colony no.	CIP resistant Colony Name	CTRL resistant Colony no.	CTRL resistant Colony Name
**CIP_BF_1**	PAO1 original ancestor	**CTRL_BF_1**	PAO1_CTRL_BF_A6_2
**CIP_BF_2**	KatA original ancestor	**CTRL_BF_2**	PAO1_CTRL_BF_A6_3
**CIP_BF_3**	PAO1_CIP A6_BFIII_2	**CTRL_BF_3**	PAO1_CTRL_BF_B6_1
**CIP_BF_4**	PAO1_CIP B6_BFIII_2	**CTRL_BF_4**	PAO1_CTRL_BF_C6_1
**CIP_BF_5**	PAO1_CIP C6_BFIII_1	**CTRL_BF_5**	PAO1_CTRL_BF_D6_3
**CIP_BF_6**	PAO1_CIPC6_BFIII_3	**CTRL_Pl_6**	PAO1_CTRL_PL_C6_1
**CIP_BF_7**	PAO1_CIP D6_BFII_1	**CTRL_Pl_7**	PAO1_CTRL_PL_C6_2
**CIP_BF_8**	PAO1_CIP A6_BFIII_3	**CTRL_Pl_8**	PAO1_CTRL_PL_C6_3
**CIP_BF_9**	PAO1CIP B6_BFIII_1	**CTRL_Pl_9**	PAO1_CTRL_PL_D6_3
**CIP_Pl_10**	PAO1CIP B6_Pl_I_2	**CTRL_BF_10**	ΔkatA _CTRL_BF_A6_1
**CIP_Pl_11**	PAO1 CIPC6_Pl_I_2	**CTRL_BF_11**	ΔkatA _CTRL_BF_B6_1
**CIP_Pl_12**	PAO1 CIP C6_Pl_I_2	**CTRL_BF_12**	ΔkatA _CTRL_BF_C6_1
**CIP_Pl_13**	PAO1 CIP C6_Pl II_1	**CTRL_BF_13**	ΔkatA _CTRL_BF_C6_2
**CIP_Pl_14**	PAO1 CIP D6_Pl_II_1	**CTRL_BF_14**	ΔkatA _CTRL_BF_C6_3
**CIP_BF_15**	ΔkatA CIP A6_BFI_2 Hp	**CTRL_BF_15**	ΔkatA _CTRL_BF_D6_2
**CIP_BF_16**	ΔkatA CIP C6_BF I_1 Hp	**CTRL_Pl_16**	ΔkatA _CTRL_PL_A6_1
**CIP_BF_17**	ΔkatA CIP D6_BFII_3	**CTRL_Pl_17**	ΔkatA _CTRL_PL_C6_1
**CIP_BF_18**	ΔkatA CIP B6_BFI_2 Hp	**CTRL_Pl_18**	ΔkatA _CTRL_PL_D6_1
**CIP_BF_19**	ΔkatA CIP A6_BFII_1		
**CIP_BF_20**	ΔkatA CIP A6_BFII_3		
**CIP_BF_21**	ΔkatA CIP B6_BFII_1		
**CIP_BF_22**	ΔkatA CIP B6_BFI_3 Hp		
**CIP_BF_23**	ΔkatA CIP B6_BFII_2		
**CIP_Pl_24**	ΔkatA CIP A6_Pl_I-1		
**CIP_Pl_25**	ΔkatA CIP B6_Pl_I_2		
**CIP_Pl_26**	ΔkatA CIP C6_Pl_I_2		

Supplementary Data Set

List of sequence variations in the 42 CIP-resistant colonies, in order of their frequencies. Sequencing reads were mapped to the reference genome of *P. aeruginosa* PAO1. The colony identity, position in the genome, coverage, frequency, gene function, description of the change in the coding region and the consequence for the encoded aminoacids, as well as the locus and gene name are presented.

Supplementary table 2. Phenotypic characteristics of the 42 CIP-resistant colonies isolated from CIP-evolved (A) and CTRL-evolved (B) populations of *P. aeruginosa* WT PAO1 and Δ*katA*. The table shows the colony morphology in light microscopy (x 1000), the colony ID of the different colonies and the characteristics of the growth curves in LB: the time of lag-phase (h), doubling time (min) and the maximum OD reached after 24 h growth. Hp indicates the hypermutable phenotype of the colony. In addition, in table S2A the fitness index (FI) for growth competition between the CIP-resistant colony and the ancestor in planktonic culture (FI plankt) for 7 or 24 hours and for growth competition in colony biofilm (FI BF) for 48 h with and without ciprofloxacin is also shown. The absence on plates of the evolved colonies after competition with the ancestor colony is marked"0".

A.

Colony morphology	Colony ID Strain/treatment/lineage /passage/experiment/col. nr	FI plankt (7h/24h)	FI BF Colony biofilm 48h vs. start without/ with cipro	Lag Phase (h)	Growth (doubling time /wt (min)	Max OD(time in hours)
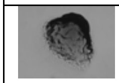	1. PAO1 AKN67 **(CIP_BF_1**)			3.5	69	1.032 (t=17)
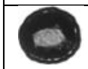	2. Δkat A AKN 67 **(CIP_BF_2)**			4.2	78	0.97 (t=16.6)
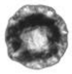	3.PAO1_CipA6_BFIII_2 **(CIP_BF_3)**	0.74	0/1.12	8.47	87.3/77	0.845 (t=19.55)
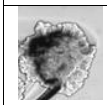	4.PAO1_CipB6_BFIII_2 **(CIP_BF_4)**	0.8	0/0	6.84	184/77	0.83 (t=17.92)
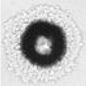	5.PAO1_CipC6_BFIII_1 **(CIP_BF_5)**	0.7	0/0.63	8.14	121/77	0.696 (t=20.85)
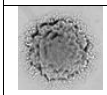	6.PAO1_CipC6_BFIII_3 **(CIP_BF_6)**	0.87	0/9.96	5.86	85/77	0.86 (t=19.22)
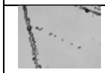	7.PAO1_CipD6_BFIII_1 **(CIP_BF_7)**	0	0/0	13	893/77	0.2254 (t=24)
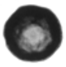	8.PAO1_CipA6_BFIII_3 (**CIP_BF_8)**	0.87	0.88/2.2	5.86	92/77	0.957 (t=18)
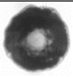	9.PAO1Cip B6_BFIII_1 (**CIP_BF_9)**	0.92	1/1.5	5.53	103/77	0.81 (t=19)
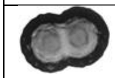	10.PAO1Cip B6_Pl_I_2 **(CIP_Pl_10)**	1.05	0.7/1.43	4.88	79.32/70.4	0.83 (t=22)
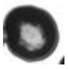	11.PAO1 CipC6_Pl_I_1 **(CIP_Pl_11)**	1.02	0/1.69	3.5	70.4/70.4	1 (t=17)
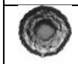	12.PAO1 Cip C6_Pl_I_2 **(CIP_Pl_12)**	0.904	0.675/1.71	3.5	70.4/70.4	1 (t=17)
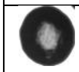	13. PAO1 Cip C6_Pl II_1	0.85	0.9/1.73	4.6	83.15/70.4	1

	**(CIP_Pl_13)**					(t=17)
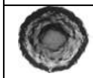	14.PAO1 Cip D6_Pl_II_1 **(CIP_Pl_14)**	1.38	0.39/1.19	5.2	67.4/70.4	0.957 (t=17.6)
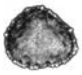	15.ΔkatACipA6_BFI_2 (Hp) **(CIP_BF_15)**	0.78	2/5.85	5.54	78.70/64	0.98 (t=16.94)
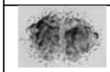	16.ΔkatA Cip C6_BF I_1 (Hp) **(CIP_BF_16)**	0.85	1.22/12	4.8	83.57/64	0.84 (t=15.96)
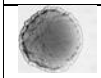	17.ΔkatA Cip D6_BFII_3 **(CIP_BF_17)**	0.86	0/9.64	4.2	64/64	0.97 (t=16.29)
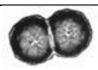	18.ΔkatA Cip B6_BFI_2 (Hp) **(CIP_BF_18)**	0.817	0/11.8	9.77	96.17/64	0.733 (t=24)
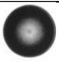	19.ΔkatA Cip A6_BFII_1 **(CIP_BF_19)**	0.64	0/0	7.5	245.5/64	0.496 (t=24)
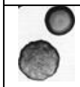	20.ΔkatA Cip A6_BFII_3 **(CIP_BF_20)**	Inhibit wt	0/1.13	5	77.87/64	0.837 (t=21.18)
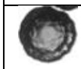	21.ΔkatA CipB6_BFII_1 **(CIP_BF_21)**	0.938	0.14/2.67	6.84	75.40/64	0.9 (t=17)
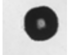	22.ΔkatA Cip B6_BFI_3 (Hp) **(CIP_BF_22)**	0.38/0.3	0/3.27	5.86	75.22/64	0.839 (t=20.8)
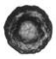	23.ΔkatA Cip B6_BFII_2 **(CIP_BF_23)**	0.55	0/0	9.7	77.42/64	0.9 (t=17)
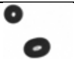	24.ΔkatA Cip A6_Pl_I_1 **(CIP_Pl_24)**	0.08/0.44	0/1.94	13.6	200/64	0.35 (t=24)
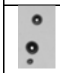	25.ΔkatA Cip B6_Pl_I_2 **(CIP_Pl_25)**	0/0.43	0	12.7	480/64	0.18 (t=24)
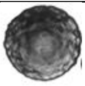	26.ΔkatA Cip C6_Pl_I_2 **(CIP_Pl_26)**	0.6/0.58	0/1.79	8.47	177.2/64	0.91 (t=21)

B.

Colony morphology	Colony identification	Lag Phase/wt (h)	Growth (doubling time/wt (min)	Max OD (time in hours)
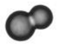	1. PAO1 CTRL BF III_A6_ 2 **(CTRL_BF_1)**	4.5/3.5	104.5/69	0.72 (t=22.5)
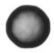	2. PAO1 CTRL BF III_A6_3 **(CTRL_BF_2)**	3.5/3.5	69	0.9 (t=17)
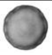	3. PAO1 CTRL BF III_B6_1 **(CTRL_BF_3)**	3.5/3.5	69	0.9 (t=17)
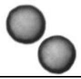	4. PAO1 CTRL BFIII_C6_1 **(CTRL_BF_4)**	3.5/3.5	69	0.9 (t=17)
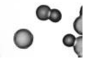	5. PAO1 CTRL BF III_D6_3 **(CTRL_BF_5)**	4.2/3.5	69	0.9 (t=17)
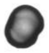	6. PAO1 CTRL Pl I_C6_1 **(CTRL_Pl_6)**	4.56/3.5	133.3/69	0.886 (t=17.9)
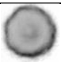	7. PAO1 CTRL Pl I_C6_2 **(CTRL_Pl_7)**	3.5/3.5	86/69	0.9 (t=17)
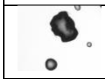	8. PAO1 CTRL Pl I_C6_3 **(CTRL_Pl_8)**	9.77/3.5	128.9/69	0.9 (t=20.8)
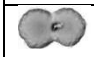	9. PAO1 CTRL Pl I_D6_3 **(CTRL_Pl_9)**	7.81/3.5	103.2/69	0.955 (t=18.89)
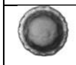	10. ΔkatA CTRL BFI_A6_1 **(CTRL_BF_10)**	5.2/4.2	115/78	0.758 (t=20)
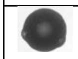	11. ΔkatA CTRL BFI_B6_1 **(CTRL_BF_11)**	5.5/4.2	114/78	0.8322 (t=20)
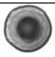 big 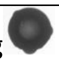 small	12. ΔkatA CTRL BFI_C6_1 **(CTRL_BF_12)**	5.2/4.2	110.5/78	0.795 (t=19.22)
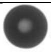 small	13. ΔkatA CTRL BFI_C6_2 **(CTRL_BF_13)**	5.2/4.2	116/78	0.789 (t=17.9)
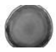 big 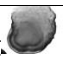	14. ΔkatA CTRL BFI_C6_3 **(CTRL_BF_14)**	4.8/4.2	90/78	0.916 (t=16.28)
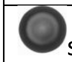 small 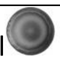 big	15. ΔkatA CTRL BFI_D6_2 **(CTRL_BF_15)**	5.8/4.2	112/78	0.835 (t=21.7)
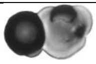 small 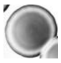 big	16. ΔkatA CTRL PlI_ A6_1 **(CTRL_Pl_16)**	6.5/4.2	131/78	0.916 (t=22.15)
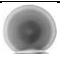	17. ΔkatA CTRL PlI_C6_1 **(CTRL_Pl_17)**	5.2/4.2	93/78	0.89 (t=22)
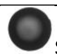 small 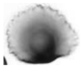 big	18. ΔkatA CTRL PlI_D6_1 **(CTRL_Pl_18)**	5.5/4.2	113/78	0.9 (t=17.59)

Supplementary table 3.

The MIC (mg/L) of the 42 CIP- resistant colonies from CIP and CTRL evolution experiments to ciprofloxacin (Cip), ceftazidime (Cefta), meropenem (Merop), aztreonam (Aztreo), tobramycin (Tobra) and colistin as determined by etest. The concentration in brackets show the antibiotic concentration at which resistant colonies (resistant subpopulations) were observed in the inhibition zone of the e-test strip. The EUCAST resistance breakpoints are presented in bold.

Colony ID<br>Strain/treatment/lineage<br>/passage/experiment/col.nr	MIC<br>Cip (**0.5**)<br>(mg/L)	MIC<br>Cefta (**8**)<br>(mg/L)	MIC<br>Merop.(**8**)<br>(mg/L)	MIC<br>Aztreo.(**16**)<br>(mg/L)	MIC<br>Tobra(**4**)<br>(mg/L)	MIC<br>Colistin (**2**)<br>(mg/L)
1. PAO1 AKN67	0.25	1	0.094	1.5	1.5	1.5
2. Δkat A AKN 67	0.125	1	0.064	1.5	1.5	1.5
3.PAO1_CipA6_BFIII_2	1 (3)	8	0.5 (1.5)	64	0.5	1
4.PAO1_CipB6_BFIII_2	1.5(3)	12	0.064 (0.5)	0.75	0.75	1.5
5.PAO1_CipC6_BFIII_1	32	8(16)	4	256	0.25-0.38	0.5
6.PAO1_CipC6_BFIII_3	0.38 (0.5)	6	0.5 (3)	24	1	1.5
7.PAO1_CipD6_BFIII_1	24	1	0.094	0.38 (0.75)	1.5	1.5
8.PAO1_CipA6_BFIII_3	8	2	0.19 (0.38)	1.5	0.75	1.5
9.PAO1Cip B6_BFIII_1	3	3	0.064	0.5-0.75	0.5	0.75
10.PAO1Cip B6_Pl_I_2	32	6	0.125(0.75)	24	1.5	1
11.PAO1 CipC6_Pl_I_1	12(32)	6	0.38(8)	96	1	1
12.PAO1 Cip C6_Pl_I_2	32	8	0.25(3)	24	1.5	1.5
13. PAO1 Cip C6_Pl II_1	32	48	0.38(8)	48	1.5	1.5
14.PAO1 Cip D6_Pl_II_1	8	6(16)	0.19(4)	48	1.5	1.5
15.ΔkatACipA6_BFI_2<br>(Hp)	32(16)	12-16(48)	0.125(0.75)	2(256)	1.5	1
16.ΔkatACip C6_BF I_1<br>(Hp)	32	2(256)	0.064(12)	1.5(256)	1.5	1.5
17.ΔkatA Cip D6_BFII_3	1(2)	4	0.094	256	1.5	1.5
18.ΔkatA Cip B6_BFI_2<br>(Hp)	32	4(12)	0.19(0.38)	3(8)	1	1
19.ΔkatA Cip A6_BFII_1	6	1.5	0.38	0.5	0.75	0.75
20.ΔkatA Cip A6_BFII_3	4(6)	2	0.064(0.094)	0.25	1	1
21.ΔkatA CipB6_BFII_1	4	24	0.094	256	1.5	1.5
22.ΔkatA Cip B6_BFI_3<br>(Hp)	32	1(2)	0.094(0.25)	256	1	1
23.ΔkatA Cip B6_BFII_2	4	2(3)	0.38	24(256)	0.5	0.5
24.ΔkatA Cip A6_Pl_I_1	3(32)	2(256)	8	256		
25.ΔkatA Cip B6_Pl_I_2	0.38(6)	8	0.094(0.5)	256	1.5	1.5
26.ΔkatA Cip C6_Pl_I_2	0.75	12	0.064(0.38)	256	1	1

1. PAO1 CTRL BF III_A6_2	1.5	1	0.032 (0.19)	0.5	0.38	0.75
2. PAO1 CTRL BF III_A6_3	4	2	0.064	0.5	0.38	1.5
3. PAO1 CTRL BF III_B6_1	2	2	0.064	1	0.75	1.5
4. PAO1 CTRL BFIII_C6_1	4(6)	2	0.064	0.5	0.5	1.5
5. PAO1 CTRL BF III_D6_3	2	3	0.064	0.75	0.75	1.5
6. PAO1 CTRL Pl I_C6_1	1.5(4)	2	0.016 (0.19)	0.75	0.75	0.75
7. PAO1 CTRL Pl I_C6_2	0.75(1)	3	0.064	1.5	1.5	1
8. PAO1 CTRL Pl I_C6_3	1(1.5)	3	0.064	1.5	1.5	1.5
9. PAO1 CTRL Pl I_D6_3	0.19(0.38)	3	0.064(0.094)	3	3	1
10. ΔkatA CTRL BFI_A6_1	3	2	0.047(0.125)	0.38	0.38	1.5
11. ΔkatA CTRL BFI_B6_1	2	1.5	0.064 (0.19)	0.5 (1.5)	0.5(1.5)	1
12. ΔkatA CTRL BFI_C6_1	2	1.5	0.047(0.125)	0.5	0.5	1
13. ΔkatA CTRL BFI_C6_2	1(3)	0.5(1.5)	0.016(0.047)	0.25 (1.5)	0.25(1.5)	1
14. ΔkatA CTRL BFI_C6_3	0.094	1.5	0.094	1.5	1.5	2
15. ΔkatA CTRL BFI_D6_2	4(8)	2	0.032	0.5	0.75-0.5	2
16. ΔkatA CTRL PlI_ A6_1	0.32(2)	3	0.064 (0.19)	2	2	1.5
17. ΔkatA CTRL PlI_C6_1	1.5(3)	1.5	0.032(0.19)	0.75	0.75	0.75
18. ΔkatA CTRL PlI_D6_1	0.064 (3)	2	0.064	2	2	1.5

Supplementary table 4.

List of mutated genes in CIP-resistant colonies isolated from both CIP and CTRL (*) biofilm (BF) and planktonic (PL) evolved populations that are correlated with the patho-adaptive genes detected in *P. aeruginosa* isolates in the CF lungs[17,18]

Colony number	Gene name
BF 3, BF 4, BF 9, BF 16, BF 18, PL 26	*retS*
BF 4, BF 5, BF 9, BF 16, BF 18, BF 19	*vgrS*
BF 5,BF 9,PL 10, BF 16, BF 18	*pil* genes
BF 3, BF 4, BF 5, BF 19, BF 20	*rpoN*
BF 3, BF 4, BF 5, BF 6, BF 9, PL 10, BF 16, BF 18, BF 19, PL 25, PL 26, *BF 1, *PL 1, *PL 7, * PL 8	*cup* genes
BF 4, BF 5, BF 16	*phuR*
BF 3, BF 4, BF 5, BF 6, BF 7, BF 8, BF 9, PL 10, BF 16, BF 17, BF 18, BF 19, BF 21, BF 25, BF 26, *BF 1, *BF 2, *BF 3, *BF 10, *BF 12, *BF 16, *PL 18	*pvd* genes
BF 5, PL 13	*ftsl*
BF 5,BF 18, PL 25, PL 26	*mutS*
BF 8, BF 15, BF 16, BF 18	*mutL*
BF 3, BF 5, BF 16, BF 18, BF 23, PL 26	*fusA1*
BF 8, BF 13,BF 21, PL 25	*aceEF*
BF 3, BF 4, BF 5, BF 6, BF 9, PL 10, BF 17, BF 19	*wsp genes*
BF 8, BF 15, BF 19, PL 24, PL 26	*morA*
BF 4, BF 8, BF 9,BF 16, PL 26	*rbdA*
BF 4, BF 7, BF 9, BF15, BF 17, BF 18, BF 19, BF 20, BF 21, PL 26, *BF 2, *BF 3, *BF 5, *PL 6, *PL 7, *PL 8, *BF 11, *BF 14, *BF 15, *PL 17	*nfxB*
BF 3, BF 5, BF 6, PL 10, PL 11, PL 12, PL 13, PL 14, BF 23, PL 25, PL 26	*mexR*
BF 23, PL 25	*nalD*
BF 6, BF 18, PL 26	*mexA*
BF 3, BF 5, BF 8, BF 9	*mexS*
BF 8, BF 11, BF 12, BF 13, BF 14	*gyrA*
BF 9, BF 10, BF 15, BF 16, BF 18	*gyrB*
